# Study protocol: A randomized, double-blind, placebo-controlled trial of isavuconazole prophylaxis for the prevention of covid-19-associated pulmonary aspergillosis

**DOI:** 10.1016/j.conctc.2024.101310

**Published:** 2024-05-17

**Authors:** Jeffrey D. Jenks, Martin Hoenigl, George R. Thompson

**Affiliations:** a Durham County Department of Public Health, Durham, NC, USA; b Division of Infectious Diseases, Department of Medicine, Duke University, Durham, NC, USA; c Division of Infectious Diseases, ECMM Excellence Center for Medical Mycology, Department of Internal Medicine, Medical University of Graz, Graz, Austria; d BioTechMed, Graz, Austria; e University of California Davis Center for Valley Fever, Sacramento, CA, USA; f Department of Internal Medicine, Division of Infectious Diseases, University of California Davis Medical Center, Sacramento, CA, USA; g Department of Medical Microbiology and Immunology, University of California Davis, Davis, CA, USA

**Keywords:** COVID-19-Associated pulmonary aspergillosis, CAPA, Invasive aspergillosis, COVID-19, Isavuconazole, Cresemba®, Antifungal prophylaxis, Opportunistic infection

## Abstract

**Background:**

During the early stages of the coronavirus disease 2019 (COVID-19) pandemic, those with severe COVID-19 infection were at risk for a number of opportunistic infections including COVID-19-associated pulmonary aspergillosis (CAPA). We initiated a randomized clinical trial to evaluate whether isavuconazole, a triazole antifungal, could prevent CAPA and improve survival in patients admitted to the ICU with severe COVID-19 infection.

**Methods:**

We designed a phase III/IV randomized, double-blind, two-arm, placebo-controlled trial evaluating standard of care (SOC) plus isavuconazole versus SOC plus placebo and were to enroll participants admitted to the ICU with severe COVID-19 infection at three medical centers in California, United States. The projected sample size was 162 participants.

**Results:**

Due to poor enrollment and the declining number of COVID-19 cases over time, the study was terminated after 7 participants were enrolled, all enrolled at one study site (UC San Diego Health). CAPA was suspected in two participants and they were started on open-label isavuconazole. One was withdrawn due to possible isavuconazole-related adverse side effects.

**Conclusion:**

Enrollment was slower-than-expected due to multiple factors, including competing COVID-19-related studies and hesitancy from potential study participants or their families to join the study. Our experience highlights some of the difficulties in planning and running a clinical trial focused on fungal superinfections involving severely ill patients during the height of the COVID-19 pandemic. Lessons learned from this study will help in the design of proposed studies examining antifungal prophylaxis against aspergillosis following other severe respiratory viral infections.

## 1 Introduction

In January 2020, a novel coronavirus termed severe acute respiratory syndrome coronavirus 2 (SARS-CoV-2) was first identified as the causative agent of pneumonia from a cluster of individuals in Wuhan City, Hubei province, China [1]. Since the first reported cases, SARS-CoV-2, which causes the disease coronavirus disease 2019 (COVID-19), has caused over 676,000,000 documented cases of COVID-19 infection and over 6,800,000 deaths as of March 10, 2023 [2].

Even before the first case of COVID-19-associated pulmonary aspergillosis (CAPA) was reported, we suspected that severe COVID-19 infection would be a predisposing factor for invasive aspergillosis (IA) [3], in part given that Influenza associated-aspergillosis has been well-documented, occurring in 16 %–23 % of influenza patients admitted to the intensive care unit (ICU) [[4], [5], [6]]. In addition, IA is an opportunistic infection associated with a number of other respiratory viral infections including parainfluenza virus and respiratory syncytial virus (RSV) [7]. The suspected pathogenesis of CAPA involves a number of immunological mechanisms, including suppression of the interferon 1 pathway and the release of danger-associated molecular patterns (DAMPs) which exacerbate the immune and inflammatory response leading to lung epithelial damage [8,9]. In addition, impairment of the innate B1a-natural IgG-neutrophil axis is thought to predispose to CAPA as well [10,11]. In one meta-analysis including 28 studies of 3184 patients admitted to the ICU with severe COVID-19 infection, CAPA occurred in 12.3 % (294/3184), with the incidence highest in central Europe and Asia [12], reflecting results of multicenter studies reporting CAPA rates between 10 % and 15 % among mechanically ventilated patients [[13], [14], [15]]. There has been one randomized clinical trial (POSA-FLU) investigating posaconazole for the prevention of influenza-associated pulmonary aspergillosis (IAPA). This study did not show a statistically significant benefit to posaconazole prophylaxis compared to no prophylaxis, although the sample size was small [16].

In April of 2020 we began planning a study evaluating isavuconazole as prophylaxis against CAPA in individuals admitted to the ICU with severe COVID-19 infection. Here we share the study design and discuss reasons why this study was ultimately terminated with lessons learned that can be applied to planned studies for aspergillosis prophylaxis in the setting of other severe viral respiratory illnesses.

## 2 Methods

This clinical trial (Isavuconazole for the Prevention of COVID-19-associated Pulmonary Aspergillosis (Isavu-CAPA)) was registered under NCT04707703 at ClinicalTrials.gov.

### 2.1 Study objectives

The primary objective of this clinical trial was to determine if anti-mold prophylaxis with isavuconazole could reduce the incidence of IA in patients admitted to the ICU with severe COVID-19 infection. The secondary objectives were to determine if anti-mold prophylaxis with isavuconazole could reduce the incidence of non-*Aspergillus* invasive fungal infections in patients admitted to the ICU with severe COVID-19 infection, improve survival from time to diagnosis of severe COVID-19 infection to time of discharge from the ICU, reduce the duration of time in the ICU compared to patients not treated with isavuconazole, reduce the duration of time on the ventilator compared to patients not treated with isavuconazole, reduce the duration of time in the hospital compared to patients not treated with isavuconazole, reduce the overall mortality rate at 30 and 90 days compared to patients not treated with isavuconazole, and determine if isavuconazole was safe in those admitted to the ICU with severe COVID-19 infection.

### 2.2 Study design

This study was a phase III/IV randomized, double-blind, two-arm, placebo-controlled trial of individuals 18 years of age or older admitted to the ICU with severe COVID-19 infection. The planned sample size was 162 participants. It was designed to provide data regarding the effectiveness of standard of care (SOC) treatment plus anti-mold prophylaxis with isavuconazole (isavuconazonium sulfate 372 mg every 8 h for 6 doses followed by intravenous isavuconazonium sulfate 372 mg once daily for up to 28 days) versus SOC plus placebo for the prevention of IA and other mold infections in the population of interest. Study sites included UC San Diego Health, San Diego, California, UC-Davis Medical Center, Sacramento, California, and UC-Irvine Health, Irvine, California, all in the United States (U.S.).

Patients admitted to the ICU with COVID-19 infection and abnormal radiographic imaging of the lungs consistent with severe COVID-19 infection were approached for inclusion in the study. COVID-19 infection occurred if there was polymerase chain reaction (PCR)-confirmed SARS-CoV-2 lab from nasopharyngeal swab (NPS), oropharyngeal swab (OPS), tracheal aspirate (TA), bronchial aspirate (BA), or bronchoalveolar lavage fluid (BALF) within 14 days prior to ICU admission or within 72 h following ICU admission. Abnormal radiographic imaging of the lungs included signs of infection (e.g. atypical pneumonia, organizing pneumonia, ground glass opacities) or ARDS that was obtained within 7 days of diagnosis of COVID-19 infection. Screening for IA was done at baseline (serum or BALF galactomannan (GM)) and in women of childbearing age a screening pregnancy test was done. If both of these tests were negative, they were deemed eligible for inclusion in the study.

If the patient or their legally authorized person (e.g. family member) consented, they were randomized to receive isavuconazonium sulfate 372 mg every 8 h for 6 doses followed by intravenous isavuconazonium sulfate 372 mg once daily for up to 28 days or SOC plus placebo every 8 h for 6 doses followed by once daily for up to 28 days. Testing for IA and other molds was done serially by fungal culture from TA thrice weekly or by serum GM thrice weekly if TA was not possible or contraindicated. Testing was done up to 28 days or until hospital discharge. Follow-up post-hospital discharge occurred monthly during the 90-day follow-up period, either by in-person evaluation or telemonitoring which was at the discretion of the study site investigator(s).

The protocol was reviewed and approved by the University of California San Diego Institutional Review Board (IRB). Both the University of California Davis and University of California Irvine were covered by the University of California San Diego IRB under the University of California IRB Reliance Registry. Informed consent forms were reviewed by the individuals IRB's at the University of California San Diego, University of California Davis, and University of California Irvine. Informed consent was obtained from all trial participants (or their legally authorized person) and their privacy rights were observed throughout the study. The study was carried out in accordance with the Declaration of Helsinki.

### 2.3 Study outcome measures

The primary study outcome was the incidence rate of IA at the time of discharge from the ICU in those who receive SOC plus isavuconazole compared to those who received SOC plus placebo. Secondary outcomes were to 1) compare the incidence of a non-*Aspergillus* invasive fungal infections at time of discharge from the ICU in those who receive isavuconazole compared to those who do not; 2) compare the rates of survival at time of discharge from the ICU in those who receive isavuconazole compared to those who do not; 3) compare the length of ICU stay in those who receive isavuconazole compared to those who do not; 4) compare the length of hospital stay in those who receive isavuconazole compared to those who do not; 5) compare the mortality rate at 30 and 90 days in those who receive isavuconazole compared to those who do not; 6) compare the rates of adverse events in those who receive isavuconazole compared to those who do not.

### 2.4 Case definition

The primary outcome, CAPA, was classified according to consensus recommendations presented by a group of experts from Europe, Asia, Africa, and North America for the diagnosis of probable and possible CAPA [17]. Probable or possible CAPA was defined as an individual with COVID-19 infection necessitating ICU-level care who had a pulmonary infiltrate or cavitating infiltrate on radiographic imaging as well as microbiologic diagnosis of an *Aspergillus* spp. infection, with the microbiologic data determining whether the case met criteria for probable or possible CAPA (Table 1).

**Table 1:** Definitions for the diagnosis of CAPA [17].

Proposed CAPA case definition (Probable)	Entry criterion: COVID-19 positive patient needing intensive care + temporal relationship	Pulmonary infiltrate, preferably documented by chest CT OR Cavitating infiltrate (not attributable to another cause)	At least one of the following:
			Microscopic detection of fungal elements in BAL indicating a mold
			AND/OR
			Serum GM index >0.5 or serum LFA index >0.5
			AND/OR
			BAL GM index >1.0 or BAL LFA index >1.0
			AND/OR
			Positive BAL culture
			AND/OR
			Multiple (>2) positive *Aspergillus* PCR in plasma, serum, or whole blood
			AND/OR
			A single positive *Aspergillus* PCR in BAL fluid (>36 cycles)
			AND/OR
			A single positive Aspergillus PCR in plasma, serum, or whole blood and a single positive in BAL fluid (any cycle permitted)
Proposed CAPA definition (Possible)	Entry criterion: COVID-19 positive patient needing intensive care + temporal relationship	Pulmonary infiltrate, preferably documented by chest CT OR Cavitating infiltrate (not attributable to another cause)	And at least one of the following:
			Microscopic detection of fungal elements in non-BAL fluid indicating a mold
			AND/OR
			Positive non-BAL culture
			AND/OR
			Single non-BAL GM > 4.5
			AND/OR
			>2 non-BAL GM index >1.2
			AND/OR
			Non-BAL GM index >1.2 plus another non-BAL mycology test positive (non-BAL, PCR, LFA)

BAL: bronchoalveolar lavage; CAPA: COVID-10-associated pulmonary aspergillosis; COVID-19: coronavirus disease 2019; CT: computed tomography; GM: galactomannan; LFA: lateral flow assay; PCR: polymerase chain reaction.

### 2.5 Sample size

While CAPA incidence rates among patients admitted to the ICU because of severe COVID-19 ranged between 19 and 35 % in studies published at the time of study initiation, the incidence in the U.S. was unknown. Assuming a conservative 15 % incidence of IA in the control arm (i.e. the lower end of the incidence range of IA in patients with severe influenza and COVID-19 infection) [[4], [5], [6],[18], [19], [20], [21], [22]] and a 2 % incidence of IA in the prophylaxis arm [23,24] we estimated that we needed a sample size of 73 in each arm and a total sample size of 146 to achieve 80 % power for detecting a significant difference between the groups (α = 0.05). Adding a 10 % loss to follow up, we estimated that we needed a total study population of 81 per arm and 162 total. If the incidence of CAPA was 20 % in the control group, the same sample size would give us at least 80 % power if the incidence in the intervention group was 4.5 % or below. If the incidence of CAPA was only 10 % in the control group, the sample size would give us 80 % power if there were no cases occurring in the intervention group.

### 2.6 Study procedures

#### 2.6.1 Participant eligibility

The inclusion criteria for the study were as follows.

1. Written informed consent obtained from the patient or their legally authorized person.
2. Adult patient (age ≥18 years).
3. Admitted to the ICU.
4. Baseline labs (including complete blood count (CBC) and liver function tests) were completed.
5. PCR-confirmed SARS-CoV-2 based on NPS, OPS, TA, BA, or BALF within 14 days prior to ICU admission or within 72 h following ICU admission.
6. Radiographic imaging consistent with SARS-CoV-2 infection (e.g. atypical pneumonia, organizing pneumonia, ground glass opacities) or ARDS within 7 days of diagnosis of SARS-CoV-2 infection.
7. A negative pregnancy test in women of child-bearing age.
8. If a woman was of child-bearing age, she had to be willing to use an effective method of contraception for 28 days after the final dose of isavuconazole, per manufacturer instructions [25].

Exclusion criteria were as follows.

1. Anticipated transfer to another medical center within hours of admission to the ICU that was not a study site.
2. Pregnancy based on a positive human chorionic gonadotropin (HCG) test from serum or urine.
3. Patient who was breastfeeding and unable to discontinue breastfeeding while taking the study drug.
4. Patients who were diagnosed with IA or detection of *Aspergillus* spp. By culture from sputum, TA, BA, or BALF or positive GM from serum or BALF at time of screening or randomization.
5. History of IA within the prior six months.
6. Known intolerance or hypersensitivity to isavuconazole or other azole agents.
7. History of familial short QT syndrome.
8. Were being treated with mold-active antifungal agents for IA or another invasive fungal infection.
9. Had severe hepatic impairment or liver cirrhosis (Child C).
10. On treatment with lopinavir/ritonavir for HIV infection.
11. Prohibited Medications
  A. Co-administration with a strong CYP3A4 inhibitor or high-dose ritonavir as they may alter the plasma concentration of isavuconazole.
  B. Co-administration with a strong CYP3A4 inducer such as rifampin, carbamazepine, St. John's wort, or long acting barbituates.
12. Prisoners or other Protected Populations

#### 2.6.2 Randomization, blinding, and study drug administration

Candidates from whom informed consent was obtained and meet the enrollment criteria were registered as participants in the study and randomized 1:1 to one of the study arms through a computer-generated randomization algorithm. Randomization was stratified for dexamethasone administration.

#### 2.6.3 Clinical evaluations

A schedule of study procedures and evaluations is outlined in Table 2. A medical history was obtained through review of the medical records and interview of the patient and/or their family members during screening. Particular attention was paid to current or prior diagnosis of invasive fungal infections, hematological malignancy, transplant status (e.g. solid organ transplant or bone marrow transplant recipient), hepatic impairment or liver cirrhosis, human immunodeficiency virus (HIV), and diabetes mellitus. Medication history included a review of all current medications including start and stop dates.

**Table 2:** Schedule of study procedures and events.

Evaluation	Screening	Prophylaxis Phase (Day)				Post Prophylaxis Phase	End of Study
		0–6	7–13	14–20	21–28		
Documentation of severe SARS-CoV-2	x						
Pregnancy test	x						
Demographics	x						
Informed Consent Obtained	x						
Baseline CBC and CMP	x						
Medical History/Medication History	x	At screening, daily while hospitalized, and post-discharge at each Clinic visit					
BALF or serum GM to screen for invasive aspergillosis	x						
Daily Isavuconazole (IV) or placebo until hospital discharge but no more than 28 days		x	x	x	x		
TDM at Day 7, 14 and Day 28 (if participant still hospitalized), ( ± 2 days)^b^			x	x	x		
TA thrice weekly for fungal culture or serum GM thrice weekly (if TA not possible or contraindicated) until hospital discharge^c^		x	x	x	x		
BALF for GM and fungal culture		If clinically indicated and performed as SOC (e.g. respiratory deterioration, persistent fever, positive serum GM)					
Chest Radiograph or Chest CT scan^a^	x	If clinically Indicated and performed as SOC (e.g. respiratory deterioration, persistent fever, positive serum GM)					
Liver Function Tests	x	As clinically indicated as SOC					
Clinical Assessment after Discharge from Hospital	x	N/A				Monthly	x

BALF: bronchoalveolar lavage fluid; CBC: complete blood count; CMP: complete metabolic panel; CT: computed tomography; GM: galactomannan; IV: intravenous; TA: tracheal aspirate; TDM: therapeutic drug monitoring; SOC: standard of care; SARS-CoV-2: severe acute respiratory syndrome coronavirus 2.

a CXR or Chest CT scan to be done as SOC as part of evaluation for severe SARS-CoV-2 infection.

b TDM level may also be obtained if participant discharged from ICU prior to Day 28 or if cytosorb, ECMO, or CCRT initiated.

c All fungal isolates, if they occur, should be sent for antifungal susceptibility testing.

#### 2.6.4 Laboratory and imaging evaluations

Laboratory evaluation included labs done at screening such as PCR-confirmed SARS-CoV-2 infection, pregnancy test, baseline CBC, complete metabolic panel (CMP) and GM from either BALF or serum to screen for current IA. Chest radiography such as a chest x-ray or chest computed tomography (CT) scan had to be consistent with COVID-19 infection (e.g. atypical pneumonia, organizing pneumonia, ground glass opacities) or ARDS within 7 days of diagnosis of COVID-19 infection. In study participants evaluation for IA was done thrice weekly by fungal culture or serum GM if culture was not possible or contraindicated. Isavuconazole therapeutic drug monitoring (TDM) was performed on Day 7, 14, and 28 of hospitalization (if participant still hospitalized). Further lung imaging with chest x-ray or chest CT scan was to be done if clinically indicated (e.g. respiratory deterioration, persistent fever, positive serum GM).

#### 2.6.5 Safety assessments

As study participants were hospitalized in the ICU, they were evaluated continuously both clinically and with daily labs, including CBC and CMP. An adverse event (AE) was defined as any unfavorable and unintended sign (including an abnormal laboratory finding), symptom, or diagnosis that occurred in a study participant during the conduct of the study regardless of the attribution (i.e., relationship of event to medical treatment/study product/device or procedure/intervention). This included any occurrence that was new in onset or aggravated in severity or frequency from the baseline condition. A serious adverse event (SAE) was defined as a death or life-threatening AE.

The study drug/placebo was held or discontinued if there was a SAE or known side effect to isavuconazole such as elevated liver laboratory tests, agranulocytosis, leukopenia, pancytopenia, acute renal failure, acute respiratory failure, hypokalemia, hypomagnesemia, and hypotension thought to be secondary to isavuconazole. If a participant developed a transaminitis, isavuconazole was discontinued immediately if one of the following occurred.

1. The ALT or AST > 8X the upper limit of normal (ULN).
2. ALT or AST remains >5X ULN over 2 weeks.
3. ALT or AST > 3X ULN & total bilirubin > 2X ULN or INR >1.5.
4. ALT or AST > 3X ULN with symptoms (e.g. fatigue, nausea and vomiting, right upper quadrant pain, fever, rash) or eosinophilia.

In addition, the study drug/placebo was discontinued if granulocytes, leukocytes, hematocrit, platelet, potassium, and magnesium levels fell below the lower limit of normal or the eosinophil count was above the ULN and these derivations were thought to be due to isavuconazole administration and not some other cause. Lastly, if creatinine clearance fell below 60 ml/min and this was sustained for at least 3 days and the cause was thought to be due to isavuconazole, then isavuconazole/placebo was discontinued.

The study participants were unblinded or withdrawn from the study if requested by the study participant, their family, or the treating medical team. In the event of a diagnosis of invasive fungal infection, including IA, the Investigator was to call the Medical Monitor who would notify the Drug Safety Monitoring Board (DSMB). In the event that all appropriate diagnostic tests had been performed to diagnose invasive fungal infection, including IA infection, a decision was made between the Medical Monitor and the DSMB as to which antifungal agent modification should be made and/or if the study patient should be unblinded if azole therapy was the only available treatment option.

### 2.7 Statistical analysis

The analysis intended to compare the proportion of study participants who developed CAPA in the intervention group versus the control group. Participants who received dexamethasone were to be stratified at randomization to the control and intervention arms given the known immunosuppressive effects of corticosteroids and risk that they may increase the chance of opportunistic infections such as CAPA. Proportions of study participants who develop CAPA in the intervention group versus the control group were to be compared using a logistic regression analysis, taking into account confounding variables including underlying medical problems, hematological malignancy and systemic corticosteroid use, and receipt of dexamethasone. It was the intention to perform a Wald test of the effect of randomized intervention using a one-sided p-value <0.05 (in the direction of improvement for the intervention group). Secondary hypotheses were to be examined by Cox proportional hazards survival analysis (for time of hospital admission to the development of CAPA) and logistic regression (for the occurrence of CAPA) using the same covariates as for the primary hypothesis and again using a one-sided p < 0.05 for hypothesis tests of the intervention effect.

## 3 Results

Although the study was to occur at three clinical sites, participants ultimately were only enrolled at one site (UC San Diego Health) prior to study termination. Eight individuals were consented to join the study. Of those, one was consented prior to the return of screening laboratory results and they were found to have a positive serum GM so were not randomized. The first participant was enrolled on April 8, 2021 and the last enrolled October 5, 2021. Given that only seven individuals ultimately were randomized, we do not discuss patient demographics or clinical outcomes here due to the risk of making the study participants identifiable. One study participant was moved to open-label isavuconazole due to clinical deterioration after 19 days in the study as the treating physicians’ desired to initiate open-label isavuconazole treatment. Another was moved to open-label isavuconazole after 7 days as *Aspergillus* spp. grew from a sputum culture and they were started on open-label isavuconazole. A third participant was withdrawn from the study due to thrombocytopenia thought to possibly be related to isavuconazole.

## 4 Discussion

Although the study was ultimately terminated due to slow enrollment, there were additional contributing issues. As with other studies performed during the COVID-19 pandemic, competitive enrollment, limited institutional resources, and enrollment during a period of significant overwork by healthcare systems and personnel limited enrollment. The IRB at the University of California San Diego was inundated with requests to perform COVID-19-related studies. Although these studies were prioritized over non-COVID-19-related studies, the approval process was still slow. In addition, since this study was funded by 10.13039/100007705Astellas Pharma Global Development Inc. (who markets and distributes Cresemba®), we were working with both Astellas and the 10.13039/100005595University of California San Diego for approval and contracting during a chaotic timeframe. In addition, the University of California San Diego developed a team of veteran investigators to assess and approve study proposals related to COVID-19 infection given the inherent limitations in the number of COVID-19-related studies that can be performed at a single institution. When institutional support was initially sought from the 10.13039/100005595University of California San Diego, we were told that this study wouldn't be prioritized as cases of CAPA had yet to be reported in the U.S. It ultimately took almost a year from the time of initial paperwork submission to the first patient being enrolled in the study. By that time much of the population was fully vaccinated against SARS-CoV-2 and hospital admissions for severe COVID-19 infection were starting to slow down. As has been discussed elsewhere, potential strategies to overcome the difficulties of performing clinical research in the setting of a pandemic may include the development and testing of pandemic-specific protocols, avoiding research competition, and developing transparent processes to select therapeutics to be studied [26].

Additionally, a number of side effects to isavuconazole are noted in the package insert, as described above, including potential isavuconazole-related side effects such as elevated LFTs, agranulocytosis, leukopenia, pancytopenia, hypokalemia, and hypomagnesemia. Given that the study participants were all critically ill in this study, a combination of laboratory abnormalities, acute renal failure, and hypotension were occurring in almost every patient. This made it very difficult for the treating medical team to determine if isavuconazole was the potential cause of these abnormalities, especially since neither they nor the Medical Monitor knew which participants were receiving isavuconazole or placebo. This resulted in the stoppage of the study drug/placebo in one study participant. Furthermore, another study participant grew *Aspergillus* spp. on culture and the treating medical team decided to unblind the participant and start them on open-label isavuconazole given the risk that they were developing CAPA and receiving placebo.

Lastly, participants were enrolled between April and October 2021. By this time – over a year into the COVID-19 pandemic – many individuals in U.S., including in California, had already completed one of the COVID-19 vaccine series, as noted above. Thus those still being admitted to the ICU with severe COVID-19 infection were largely unvaccinated. There were a number of reasons why individuals were not vaccinated against COVID-19 during the pandemic, including inequities in vaccine distribution, historical health inequities and disenfranchisement of certain populations, difficulties accessing vaccines, and vaccine hesitancy [27]. The reasons that contributed to vaccine hesitancy may also have contributed to hesitancy to enroll in this study as we found that a lot of potential study participants or their family members declined enrollment in this study, which contributed to slow accrual.

In terms of statistical analysis, logistic regression was proposed for evaluating the effect of the randomized intervention using a one-sided p-value <0.05 (in the direction of improvement for the intervention group). In retrospect, given that COVID-19 has high mortality in severely ill patients, and death is a competing risk if the outcome of interest is development of CAPA, competing risk analysis might appear more appropriate.

Since termination of our trial, a number of single center observational studies have reported on the benefits of antifungal prophylaxis in preventing CAPA [[28], [29], [30]], and while still no RCT has been performed on CAPA prophylaxis, one RCT evaluating prophylaxis with inhaled opelconazole is currently in the planning phase [31,32]. In addition, a recent single-center study found a high incidence of CAPA in patients admitted to the ICU in in the vaccination era, and there are calls to investigate antifungal prophylaxis in this population [33].

In conclusion, here we present the study design for a randomized, double-blind, two-arm, placebo-controlled trial of individuals 18 years of age or older admitted to the ICU with severe COVID-19 infection. We sought to evaluate whether SOC plus isavuconazole could reduce the incidence of IA and other mold infections and improve survival, reduce ICU stay, reduce time on ventilator, reduce duration of hospitalization, and improve survival in patients admitted to the ICU with severe COVID-19 infection, compared to placebo. We encountered difficulty keeping study participants blinded given that they were all severely ill with multiple laboratory abnormalities and it was difficult to rule out that isavuconazole was the potential cause, even though among the azole antifungal isavuconazole is often considered one of the safest and best-tolerated. In addition, a number of study participants clinically deteriorated and were given open-label isavuconazole. Ultimately, due to slow enrollment the study was terminated. We are hopeful that this study design and lessons learned from this study will help in the design of proposed studies examining antifungal prophylaxis against aspergillosis following COVID-19 or other severe respiratory viral infections such as influenza, parainfluenza, and RSV given the ongoing risk of opportunistic IA in at-risk populations.

## Funding

This study was funded by 10.13039/100007705Astellas Pharma Global Development, Inc.

**Table:** Abbreviations

AE: adverse event	HIV: human immunodeficiency virus
ALT: alanine transaminase	ICU: intensive care unit
AST: aspartate aminotransferase	IA: invasive aspergillosis
ARDS: acute respiratory distress syndrome	IRB: Institutional Review Board
BA: bronchial aspirate	LFTs: liver function tests
BALF: bronchoalveolar lavage fluid	NPS: nasopharyngeal swab
CAPA: COVID-19-associated pulmonary aspergillosis	OP: oropharyngeal swab
CBC: complete blood count	PCR: polymerase chain reaction
COVID-19: coronavirus disease 2019	RSV: respiratory syncytial virus
CMP: complete metabolic panel	SAE: severe adverse event
CT: computed tomography	SARS-CoV-2: severe acute respiratory syndrome coronavirus 2
DSMB: drug safety monitoring board	SOC: standard of care
GM: galactomannan	TDM: therapeutic drug monitoring
HCG: human chorionic gonadotropin	ULN: upper limit of normal

## CRediT authorship contribution statement

**Jeffrey D. Jenks:** Writing – original draft, Supervision, Resources, Project administration, Methodology, Investigation, Funding acquisition, Formal analysis, Conceptualization. **Martin Hoenigl:** Writing – review & editing, Methodology, Investigation, Funding acquisition, Conceptualization. **George R. Thompson:** Writing – review & editing, Methodology, Investigation, Funding acquisition, Conceptualization.

## Declaration of competing interest

The authors declare the following financial interests/personal relationships which may be considered as potential competing interests:

This study was funded by 10.13039/100007705Astellas Pharma Global Development, Inc. All authors have received funding from Astellas but they did not influence the work reported in this paper.
